# The Reactions of Photosynthetic Capacity and Plant Metabolites of *Sedum hybridum* L. in Response to Mild and Moderate Abiotic Stresses

**DOI:** 10.3390/plants11060828

**Published:** 2022-03-21

**Authors:** Nina V. Terletskaya, Gulnaz A. Seitimova, Nataliya O. Kudrina, Nataliya D. Meduntseva, Kazhybek Ashimuly

**Affiliations:** 1 Faculty of Biology and Biotechnology and Faculty of Chemistry and Chemical Technology, Al-Farabi Kazakh National University, Al-Farabi Avenue 71, Almaty 050040, Kazakhstan; sitigulnaz@mail.ru (G.A.S.); nat.mdnt@gmail.com (N.D.M.); kajeke@mail.ru (K.A.); 2 Institute of Genetic and Physiology, Al-Farabi Avenue 93, Almaty 050040, Kazakhstan

**Keywords:** water deficit, salt stress, cold stress, photosynthesis, secondary metabolites

## Abstract

In this article, for the first time, an experimental study of the effect of mild and moderate osmotic stress, NaCl content and the effect of low positive temperature on photosynthetic activity and composition of metabolites of immature plants *Sedum hybridum* L. is reported. In this representative of the genus *Sedum* adapted to arid conditions and having the properties of a succulent, a change in photosynthetic activity and an increase in the level of protective metabolites in the shoots were revealed when exposed to mild and moderate stress factors. The results of this study can be used in work on the adaptation of succulent plants to arid conditions, environmental monitoring and work on the directed induction of valuable secondary metabolites in succulents to obtain new herbal medicines.

## 1. Introduction

Literature data indicating global climate change on Earth anticipate the possible degradation and desertification of more than 90% of territories by 2050, portending an ecological catastrophe [1]. The literature data indicate that the aridization of the climate influences many metabolic processes in plants [2,3,4].

Plants in the natural environment are exposed to a wide range of stress effects of different intensities such as mild stress, moderate stress or severe stress, which results in dehydration, wilting and, ultimately, the death of the plant. The literature shows that plants use different strategies to deal with stresses of varying intensity [5,6,7]. In general, researchers create extreme stress in laboratory experiments, although in field conditions, plants often experience a moderate effect of abiotic stresses [8]. In this context, the effect of mild and moderate abiotic stresses on plants physiologically and anatomically adapted to arid conditions, in particular succulents, is of distinct interest.

This group includes plants of the genus *Sedum* of the *Crassulaceae* family, rich in valuable biologically active substances, including up to 600 species, the growth and survival of which in conditions of abiotic stressful effects are still insufficiently understood.

Plants develop effective mechanisms such as water balance regulation, stomatal conduction, transpiration, cell wall architecture, membrane structure remodeling, cell cycle changes and light absorption regulation, photosynthetic activity, and others that determine both growth processes and precise physiological adaptation settings [9] to survive and evolve in complex and ever-changing environmental conditions. In the process of adaptation, the body can rebuild, reach a new homeostatic level, activate some physiological systems or slow down others.

To study the reactions of a plant organism to environmental stresses, the photosynthetic activity of leaves is a very important criterion, since it directly contributes to plant growth and productivity under the action of abiotic stressors [10,11]. Photosynthesis is a process in which the energy of light is used to produce ATP and NADPH, which are eventually consumed to assemble carbon atoms in organic molecules [12]. It is known that under most abiotic stresses, even with mild and moderate stress, the absorbed light energy exceeds the required limit of use by the plant. Consequently, it leads to the formation of reactive oxygen species, such as superoxide-anion radical (O^−^_2_), hydrogen peroxide (H_2_O_2_) and singlet oxygen ^1^O_2_ [13,14,15]. When reactive oxygen species (ROS) production prevails over an antioxidant defense network, photo oxidant stress occurs [13,16,17]. Therefore, when faced with changes in environmental conditions, plants first need to regulate photosynthetic electron transport to prevent the accumulation of damaging oxygen by-products [18]. Meanwhile, the change in photosynthesis is a consequence of the most painful processes, which are affected by environmental stress factors that cause an increase in the level of stress in plants.

Biosynthesis and the accumulation of a vast and very diverse range of biologically active organic compounds in plants are mainly triggered by light [19,20]. These substances are commonly referred to as secondary metabolites (SMs). They are distributed differently among different taxonomic groups in the plant kingdom, and the vast majority of them are not immediately involved in plant growth and development [21]. Secondary plant metabolites, which are often synthesized in response to environmental stresses, partly determine the ability of plants to survive and adapt to adverse environmental conditions. Changes in photosynthetic activity and the accumulation of secondary metabolites combine in plant responses to oxidative stress that develop under the influence of adverse environmental factors [17,22,23].

In contrast to primary metabolism, which is responsible for the main mechanisms of synthesis, secondary metabolism is of particular importance not for each cell, but for the entire plant organism as a whole [24]. Meanwhile, changes in the pattern of the accumulation of secondary metabolites in stressful conditions for the plant are the result of differences in both the rate of synthesis and the rate of catabolism of primary compounds [25,26]. The stress defense responses of the plant are coordinated by signaling processes that trigger primary metabolism and provide biosynthetic products for secondary metabolism. Therefore, plants synthesize large amounts of SMs, and the production of these metabolites manifests as an adaptive ability to cope with the adverse effects of various environmental stresses [27,28].

Nowadays, plant SMs have become the subject of sharply growing interest both in connection with their importance for medical, nutritional and cosmetic purposes, and for their indisputable value for the plants themselves with their role in the physiology of stress [29,30,31,32]. Many SMs, having antioxidant properties, ensure the survival, stability and competitiveness of plants in adverse conditions. It was revealed that it is the increasing content of SMs, under the influence of various stressors, that triggers signaling functions for the regulation of genes responsible for protecting the plant organism [28]. Therefore, the study of SMs as a function for protecting plants from biotic and abiotic stresses is becoming increasingly essential [33,34]. In addition to the protective functions of the whole plant, SM molecules also act as protection of primary metabolites, such as proteins and nucleic acids, from stress-induced damage [35,36].

Consequently, secondary metabolism in the plant organism is responsible for many physiological modifications along with the regulation of ionic and water balance, minimization of oxidative damage, etc., which can directly or indirectly provide the plant’s phenotypic response to stress.

*Sedum hybridum* L. (*Aizopsis hybrid* (L.) Grulich) is a perennial herbaceous plant forming low mats 8–15 cm tall, with a long, branchy, woody, creeping rhizome and thin roots. It blooms from June, bears fruit in July–August. This winter-hardy and drought-resistant, relatively slow growing sedum is not demanding on the soil [37]. The species is widely distributed in the Tien Shan foothills. The shoots of *S. hybridum* are used as a hemostatic, antiseptic, antibacterial, laxative, diuretic and tonic in folk medicine, and therefore deserves phytochemical, pharmacological and clinical study. However, despite the various pharmacological effects of this species, the phytochemical composition of *S. hybridum* is practically unstudied [38,39].

The purpose of this work was to experimentally study the effect of abiotic stresses, such as mild and moderate osmotic stress, increased NaCl content and the effect of low positive temperature on the morphophysiological parameters and composition of secondary metabolites of immature plants *S. hybridum*. Mild and moderate osmotic, salt and cold stresses are the main stresses that *S. hybridum* faces in its natural environment. Therefore, the stress modes of exposure we chose did not pursue the goal of studying the sensitivity threshold of the drought- and salt-tolerant succulent *S. hybridum* but were aimed at obtaining more detailed information about its adaptive mechanisms. Analysis of the results will reveal both changes in the physiological state of plants of this species in detrimental conditions and shifts caused by abiotic stress in the content of certain classes of secondary metabolites in the shoots of this succulent. These stress factors are well modeled in the laboratory and, in the future, can be used for targeted stimulation of medicinally valuable secondary metabolites. We hope that our information will be valuable for better understanding of the mechanisms of protection against adverse conditions, approaches to the targeted synthesis of valuable secondary metabolites and optimization of the possibility of obtaining new phytopreparations.

## 2. Results

### 2.1. Comparative Analysis of the Water Content and Photosynthetic Parameters of S. hybridum in Different Conditions

A comparison of the immature plants of the succulent *S. hybridum* showed no significant differences in biomass and water content in the leaves of the stressed immature plants compared to the controls (Table 1).

**Figure 1:**
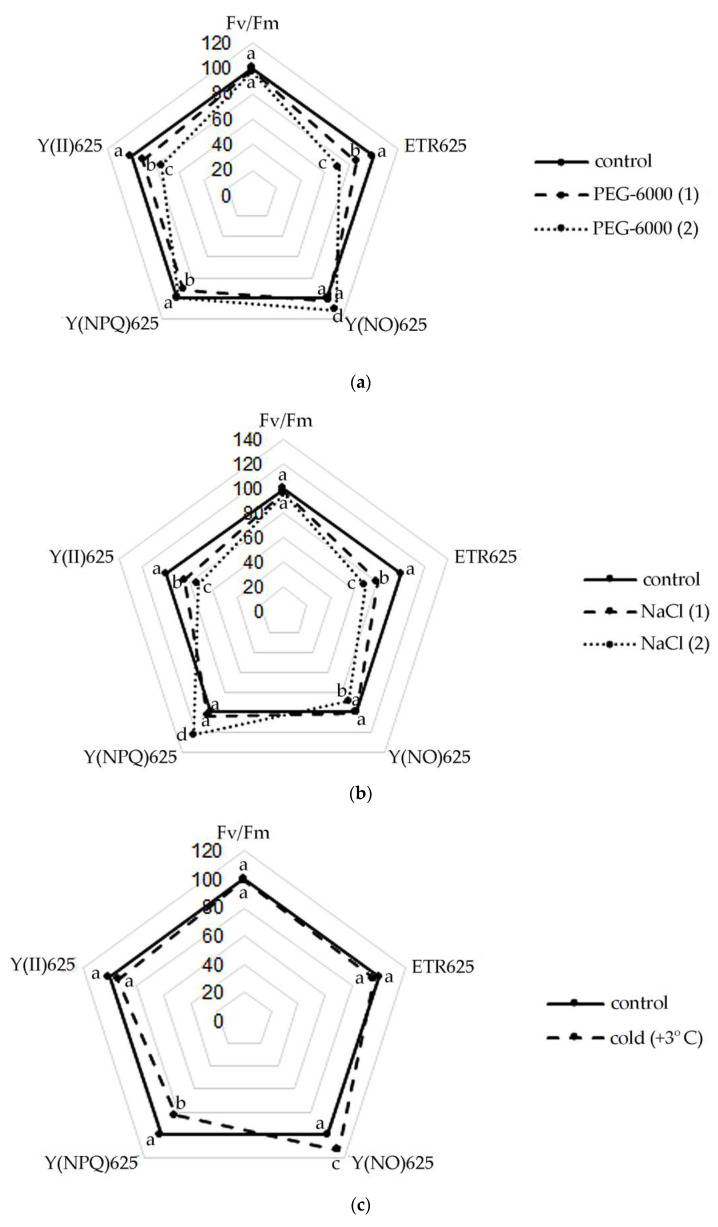
Changes in the activity of the photosynthetic apparatus of *S. hybridum* under stress conditions: (**a**) osmotic stress; (**b**) salt stress; (**c**) cold stress. Different letters above the peaks of the pentagram represent significant differences at *p* ≤ 0.05, *n* = 3 plants in each of 3 replicates for all treatments.

However, the data presented in Figure 1 show that *S. hybridum* plants exposed to stress, despite the absence of differences from the control of water content in the leaves, still experienced physiological stress, expressed in changes in the operation of the photosynthetic apparatus.

Thus, a slight decrease in the Fv/Fm ratio was noted in the leaves of *S. hybridum* with an increase in the stressor concentration (Figure 1a–c). Meanwhile, the observed changes in this parameter were not statistically significant. A similar picture was obtained in terms of the change in the effective quantum yield of PhS II at PPFD 625 µmol m^−2^ s^−1^, with the difference that the effect of increased concentrations of osmotic and salt stressors was statistically significant for this photosynthetic parameter.

It was also found that the rate of non-cyclic electron transport through PhS II (ETR) decreased in all variants of the experiment, except for the form of cold exposure. The decline in ETR values increased at the level of both osmotic and salt stress.

An upward trend in Y(NO) was also shown under osmotic stress and cold. However, with increasing NaCl concentration, an interesting phenomenon of declining Y(NO) in the leaves of *S. hybridum* was noted (Figure 1b). A high level of values of the quantum output of adjustable energy dissipation Y(NPQ) at PPFD 625 µmol m^−2^ s^−1^ in the presence of a stressor was also revealed (Figure 1b).

### 2.2. Comparative Qualitative Analysis of the Biologically Active Substances of S. hybridum in Different Conditions

Preliminary phytochemical screening was carried out using various chemical assays in order to identify major classes of biologically active compounds in extracts from *S. hybridum* (Table S1). Qualitative analysis of plant metabolites found in extracts under control and stress conditions made it possible to determine certain patterns. Thin-layer chromatography (TLC) in different solvent systems using specific developers was used to monitor the identity of the qualitative content of each of the extracts; the groups of the biologically active compounds of studied samples were screened and examined which revealed that the major groups are flavonoids, phenolic compounds, tannins, terpenoids, amino acids and carbohydrates.

A comparative analysis of the variants subjected to stress influences made it possible to assert that the studied samples were different in their contents of the main classes of SMs or groups of substances. Phenolic compounds were also quite abundant in all extracts, possibly in the form of tannins, which was evidenced with a solution of ferric chloride, a gelatin test and lead acetate.

All samples in the following solvent systems: chloroform: methanol: water (4:3:1), chloroform: methanol: acetone (8:1:1) and n-butanol: acetic acid: water (10:3:7), showed the presence of phenolic compounds, which were visualized under UV light at 365 nm (R*_f_* values 0.51, 0.64, 0.65). The presence of steroids and terpenoids (pink/purple spots) was detected by spraying the TLC plates with vanillin–sulfuric acid followed by heating (R*_f_* value 0.39). Terpenoids and steroids tests were showed positive for all extracts.

Meanwhile, it was noted that the content of phenols, phenolic acids, flavonoids and quinones was higher in samples subjected to moderate osmotic and moderate salt stress. Control samples, as well as samples exposed to low positive temperature or moderate osmotic stress, contained amino acids, tannins, flavonoids and carbohydrates in the greatest quantities. High contents of terpenes and their glycosides were found in the controls and samples exposed to a low positive temperature. Mild and moderate osmotic stress samples were strongly positive to Wagner’s and Dragendorff’s tests observing an orange precipitate, whereas other extracts showed a complete absence of alkaloids. The general reagent for selectively detecting alkaloids is Dragendorff’s reagent (orange–brown spots), color reaction on developed TLC plate showed the presence of alkaloids in hexane: ethyl acetate: methanol: water (6:12:4:1), two spots were obtained with R*_f_* of 0.16 and 0.95.

### 2.3. Comparative Quantitative Analysis of the Biologically Active Substances of S. hybridum in Different Conditions

**Figure 2:**
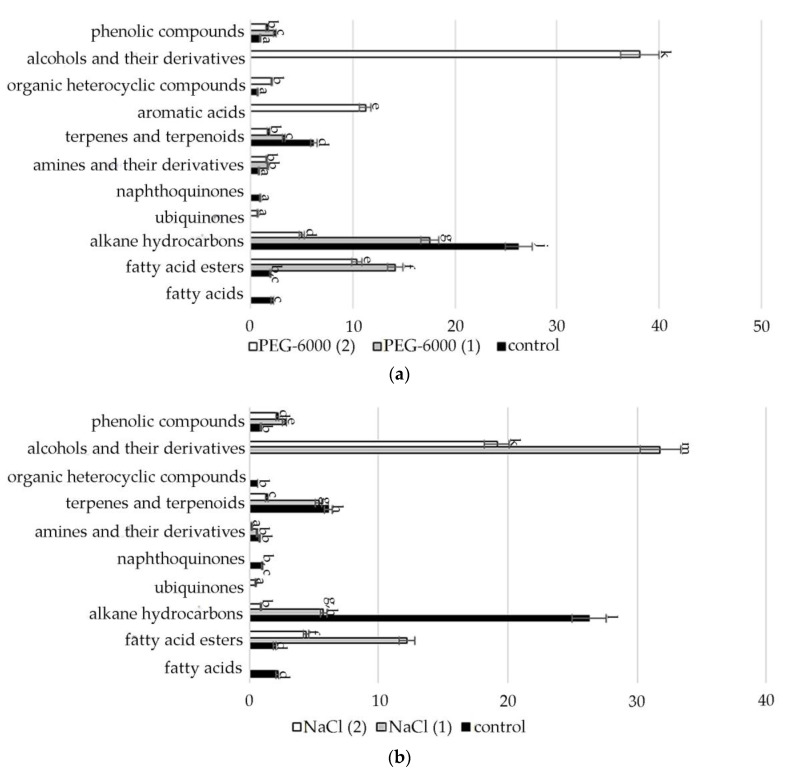

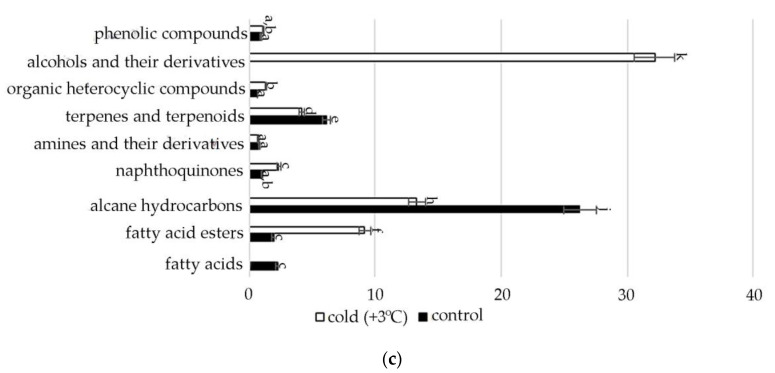
Change in content of secondary metabolite classes in *S. hybridum* under stress conditions: (**a**) osmotic stress; (**b**) salt stress; (**c**) cold stress. Values presented are means (±SD). Different letters above the bars represent significant differences at *p* ≤ 0.05, *n* = 3 plants in each of 3 replicates for all treatments.

A comparative quantitative analysis of the GC-MS spectrum of biologically active compounds of control group plants with the spectrum of *S. hybridum* shoots cultured under mild and moderate osmotic stress (Figure 2a) demonstrated the disappearance of compounds such as fatty acids and naphthoquinones, and the appearance of compounds such as fatty acid esters, γ-tocopherol, aromatic acid, alcohols and their derivatives, (Figure 2a, Table S2).

Data obtained under the conditions of mild and moderate salt stress (Figure 2b, Table S2) present the absence of fatty acids, naphthoquinones, aromatics of acids and organic heterocyclic compounds.

In the spectra of *S. hybridum* shoots cultured under the influence of a low positive temperature (Figure 2c, Table S2), the presence of all compound classes was detected, except for fatty acids and aromatic acid.

**Figure 3:**
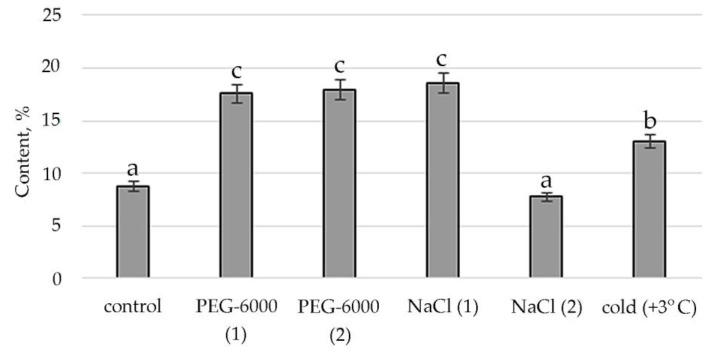
Change in content of hexadecanoic acid, ethyl ester in *S. hybridum* under stress conditions. Values presented are means (±SD). Different letters above the bars represent significant differences at *p* ≤ 0.05, *n* = 3 plants in each of 3 replicates for all treatments.

**Figure 4:**
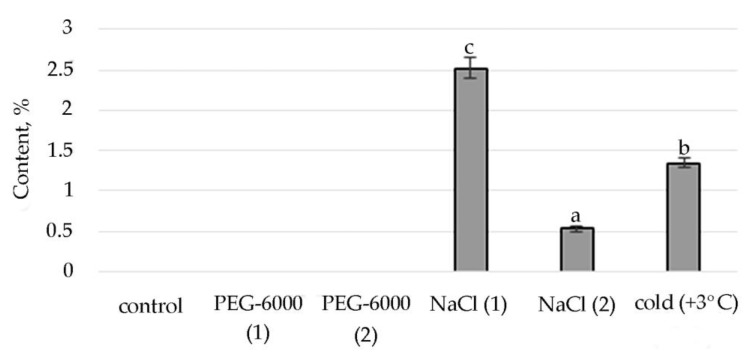
Change in content of tetratetracontane in *S. hybridum* under stress conditions. Values presented are means (±SD). Different letters above the bars represent significant differences at *p* ≤ 0.05, *n* = 3 plants in each of 3 replicates for all treatments.

**Figure 5:**
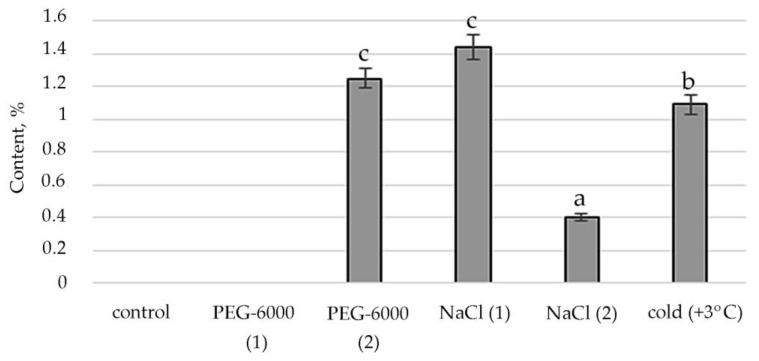
Change in content of phytol in *S. hybridum* under stress conditions. Values presented are means (±SD). Different letters above the bars represent significant differences at *p* ≤ 0.05, *n* = 3 plants in each of 3 replicates for all treatments.

**Figure 6:**
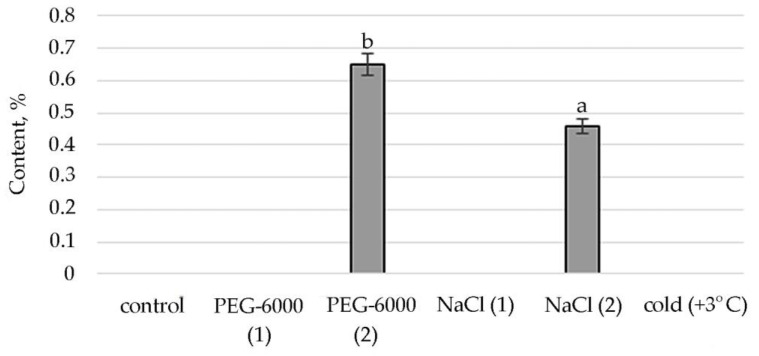
Change in content of γ-tocopherol in *S. hybridum* under stress conditions. Values presented are means (±SD). Different letters above the bars represent significant differences at *p* ≤ 0.05, *n* = 3 plants in each of 3 replicates for all treatments.

**Figure 7:**
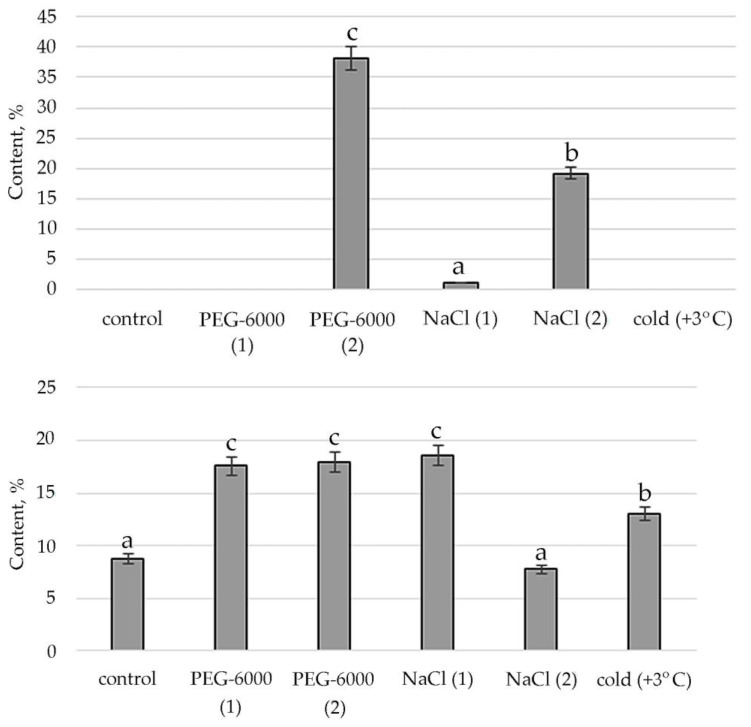
Change in content of 1,2,4-Butanetriol in *S. hybridum* under stress conditions. Values presented are means (±SD). Different letters above the bars represent significant differences at *p* ≤ 0.05, *n* = 3 plants in each of 3 replicates for all treatments.

**Figure 8:**
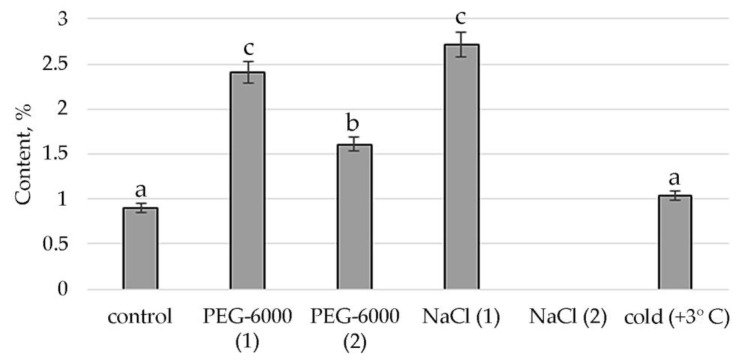
Change in content of 1,4-Benzenediol, 2,5-bis(1,1-dimethylethyl)- in *S. hybridum* under stress conditions. Values presented are means (±SD). Different letters above the bars represent significant differences at *p* ≤ 0.05, *n* = 3 plants in each of 3 replicates for all treatments.

Meanwhile, within almost all the metabolite classes, changes in the spectral composition were revealed both in the presence/absence of individual components and in the increase/decrease in their quantitative contents under the influence of abiotic stresses. The data are presented in Figure 3, Figure 4, Figure 5, Figure 6, Figure 7 and Figure 8.

Therefore, according to the structural group composition of the ethanol extract of *S. hybridum* shoots on stress backgrounds, a change in the content of hexadecanoic acid, ethyl ester—an ester of fatty hexadecanoic acid (Figure 3) was observed. The quantitative content of these components has a clear tendency to rise with increasing osmotic stress, as well as a boost and decline with increased salt stress and an almost unchanged state in relation to control under the influence of low positive temperature.

The synthesis of the biologically active substance Tetratetracontane contributes to mild salt stress. With an increase in stress exposure, the percentage of this SM decreases sharply. Some stimulating effects on the synthesis of Tetratetracontane in *S. hybridum* can be observed under the influence of a low positive temperature (Figure 4).

The stimulating effect of moderate osmotic, mild salt stress and low positive temperature on Phytol, an acyclic aliphatic organic chemical compound, a single-unsaturated diterpene which is part of vitamins E and K1, was noted (Figure 5).

Conditions of moderate osmotic and moderate salt stress in the shoots of *S. hybridum* stimulate the synthesis of a representative of ubiquinone’s γ-tocopherol, a fat-soluble vitamin E (Figure 6).

It was revealed that the synthesis of alcohols and their derivatives in the shoots of *S. hybridum* is also vital due to the influence of abiotic stress factors. Figure 7 shows the peaks of its quantitative content when exposed to moderate osmotic and salt stress.

Finally, the response of phenols and their derivatives to mild and moderate stress is shown in Figure 8. In the case of 1,4-Benzenediol, 2,5-bis(1,1-dimethylethyl), peaks in the content of this metabolite are detected in mild osmotic and salt stress. Moderate osmotic stress also has some stimulating effect on its synthesis, while the low positive temperature leaves the presence of this substance almost at the level of control conditions.

## 3. Discussion

Our data show that in the absence of visible changes in leaf turgor, physiological and biochemical changes still occur, caused by stress effects. They are aimed primarily at maintaining the vital activity of plants but can also be practically valuable for humans.

Among the fluorescence parameters of chlorophyll used to monitor adaptation to stressful influences, the indicator of a decrease in the maximum efficiency of PS II photochemistry (Fv/Fm) is most frequently mentioned in the literature [40]. In general, the more stress the plant experiences, the fewer open reaction centers are available in its PS II. However, some researchers question the effectiveness of this parameter as a good indicator of stress resistance [11,40,41]. It is clear that the increase in the sensitivity of the leaf to photoinhibition under stressful conditions is expressed in a reduction in the Fv/Fm ratio, which we also observed in *S. hybridum* with a growth in the concentration of the stressor. However, the data obtained for this parameter in our experiment were not statistically reliable compared to the control and did not give a clear picture of the response of *S. hybridum* shoots to the effects of mild and moderate abiotic stress.

Plants have various mechanisms for dissipating excess energy to protect and optimize photosynthesis under adverse conditions [18]. The literature describes various fluorescence-based parameters for quantum outputs of non-photochemical energy conversion to PS II, Y(NPQ) and Y(NO) that complement the quantum yield of the photochemical energy conversion, Y(II) [42]. In accordance with this, our results obtained from the last pulse of the light curve at 625 µmol m^−2^ s^−1^, demonstrating a decrease in the values of Y(II) and ETR values in plants under all stress effects experienced, can be associated with activation of non-photochemical extinguishing mechanisms, while relatively high values of electron transport rates can serve as an indicator of the normal level of photosynthetic activity [43]. An increase in the level of Y(NO) under moderate water stress and cold stress (Figure 1a,c) also clearly indicates problems with the redistribution of excess light energy entering PS II [44,45]. However, with an increase in the concentration of NaCl, an interesting phenomenon of a decrease in Y (NO) in the leaves of *S. hybridum* was noted, which is a sign of the absence of disturbances in the work of PS II in *S. hybridum* with increased salt stress (Figure 1b).

The high level of the quantum yield of controlled energy dissipation Y(NPQ), which we noted at PPFD 625 µmol m^−2^ s^−1^ in the presence of a stressor (Figure 1b), in comparison with the control, indicates the maximum activation of PS II defense mechanisms. In this case, it confirms the high level of salt tolerance of *S. hybridum*, and demonstrates its good defense mechanisms against the effects of osmotic stress (Figure 1a). We can assert this based on literature data that NPQ is involved in the mechanism of plants adaptation to biotic or abiotic stress and is a major component of systemic acquired resilience [46,47]. A higher NPQ indicates an adaptive response of plants to increased dissipation of absorbed energy in heat form to protect their thylakoid membranes from photo damage and can compensate for the decrease in Y(II) and even cause a lowering in Y(NO) [48]. Leaves activate NPQ to dissipate excess excited energy in the form of heat and regulate the photosynthetic flow of electrons during the induction of photosynthesis, as well as to avoid the harmful formation of ^1^O_2_, which can damage the photosynthetic apparatus [49,50]. Therefore, the leaf can maintain a balanced ROS level that ensures growth and prevents oxidative damage by adjusting the NPQ [47,51,52,53].

An important line of defense against various stressful influences are plant metabolites. While the removal of the anion radical (O^−^_2_) is mainly achieved by an antioxidant enzyme, superoxide dismutase [17], singlet oxygen ^1^O_2_ can only be controlled by non-enzymatic plant antioxidants [22,23].

Despite the fact that most of the literature suggests the adaptive mechanisms of plants to the action of stressors largely depend on the functions of non-volatile antioxidant compounds, our analysis of mass spectra showed that stressful conditions significantly alter the dominant phytochemical spectrum of other shoot metabolites of *S. hybridum*, which also play significant roles in protecting against adverse environmental conditions [54,55,56].

Accordingly, in our experiment, the fatty acids (FAs) themselves were present only in the mass spectrum of control plants. However, in the mass spectrum of plants exposed to stress, there was an increase in a variety of FA esters. FAs are the main components of all plant cells [57]. If exposure to abiotic stressors causes a violation of the conductivity of the bio membrane, plasma membrane fluidity correlates with the proportion of unsaturated fatty acids [58,59]. In addition, FA serve as precursors of the main plant hormones, such as jasmonates, which are involved in the process of adaptation to stress [60]. The increased content of FAs, including in the form of various esters, in the lipids of the inner membranes of chloroplasts and mitochondria, can correlate with the weakening of photoinhibition of PS II [61]. To a certain extent, this confirms the greater quantities of FA-esters at mild than at moderate abiotic stress (Figure 3, Table S2).

Lipid metabolites, in addition to FAs and their esters, also include their reduced forms, including aldehydes, alkanes, ketones and alcohols, often part of cuticular waxes [62,63]. Varieties of alkane hydrocarbons, according to the literature, are widely produced by plants growing in areas with insufficient water supply [64,65]. In our experiment, an increased biosynthesis of some lipophilic alkane hydrocarbons, such as eicosane, heneicosane, tetracosane (with mild osmotic stress) (Figure 4), nonacosane (with moderate osmotic stress), pentacosane (with mild osmotic and mild salt stress), tetratetracontane (with mild and moderate salt stress and under the influence of low positive temperature) was revealed (Figure 4, Table S2). These substances, according to the literature data, can be included in the composition of epicuticular waxes in representatives of the genus *Sedum* and quantitatively depend on the type of habitat [66]. Virtually all of these substances possess antioxidant, antimicrobial, anti-inflammatory, analgesic, antipyretic and other effects and can be used to develop drugs in pharmacology and medicine [67,68,69].

Lipid metabolites are also hydrocarbons that are biosynthesized either from FA or from terpenoids in a direct way. Moreover, the terpenoids themselves contribute greatly to lipid metabolism and accumulation [70]. We noted the absence of a decrease in the concentration of terpenes under mild salt stress. Boncan et al. (2020) pointed to the importance of terpenoids for protecting plants: Under abiotic stress, volatile terpenes mitigate the effects of oxidative stress, either through direct intercellular reactions with the oxidant or by altering ROS signaling [71]. The amphipathic nature of terpenes enhances the hydrophobic interactions between membrane proteins and lipids [72], which prevents membrane breakdown and protein breakdown. The singlet oxygen produced by oxidative stress is also removed by the terpenoids [73]. Additionally, the literature shows a significant role of phytol, which is part of chlorophyll, vitamin E and vitamin K₁, in resistance to high temperatures and prolonged exposure to light [74]. The results of our experiments demonstrate the active biosynthesis of phytol under mild and moderate salt stress and exposure to low positive temperature (Figure 5).

The identified presence of γ-tocopherol in the mass spectra of plants subjected to moderate stress (both osmotic and saline) and the increase in the concentration of 2-Anilino-1,4-naphthoquinone in relation to the control under conditions of cold exposure confirms that quinones act as antioxidants in oxidative reactions. This is confirmed by literature data that quinones, which have a labile hydroxyl group and relatively easily react with hydrocarbon peroxide radicals, inhibit the initial stage of oxidative stress in cell membranes. In particular, tocopherol, which in response to tocopherol abiotic stress, mediates several retrograde signaling pathways associated with hormones, reactive oxygen species, and microRNAs [75,76,77].

We discovered that phenolic compounds, such as 1,2,3-Benzenetriol phenolic acid and 1,4-Benzenediol, 2,5-bis(1,1-dimethylethylethyl) (Figure 8, Table S2) showed increased synthesis under the stress conditions of our experiment, which is consistent with literature data about the fact that their synthesis is enhanced in response to external stimuli [78,79]. Gill & Tuteja (2010) noted that during a stressful state, plants become potentially active and, by releasing a phenolic substance modulating antioxidant and enzyme and radical scavenging activity, are actively involved in combating oxidative stress [13]. The presence of three phenolic compounds was previously identified in a member of the *Sedum* genus, *Sedum sarmentosum* Bunge (*Crassulaceae*) [80], the synthesis of which is determined by the influence of the environment. They take part in photosynthesis, formation of lignin and suberin and are, like anthocyanin, plant pigments [81]. Their phenolic compounds can affect respiration and oxidative phosphorylation, alter membrane permeability, etc. [79]. They are involved in suppressing or blocking free radicals, the most characteristic lipid peroxidation (LP) reaction, and generally have cytoprotective effects [41]. This fact also confirms their importance in adaptive responses under mild and moderate abiotic stresses.

Furthermore, all the amines isolated in our experiment showed increased content in one way or another under stressful conditions in relation to control (Table S2). This is explained by literature data suggesting that amines, due to their positive charges, bind to reactive oxygen species (ROS). Thus, stabilizing the plasma membrane (PM), and polyamine-modulated activity or expression of H^+^-ATPase of the plasma membrane increases the tolerance of plants to adverse environmental factors [82].

All this implies that abiotic stress effects, even in mild and moderate forms, negatively affect plants. Even succulents, which seem to be adapted to arid conditions, to which *S. hybridum* belongs, cause protective reactions of the plant organism, changing photosynthetic activity and increasing the production of protective metabolites in shoots.

## 4. Conclusions

The paper describes a detailed study on the effect of osmotic and salt stress in mild (−0.6 MPa) and moderate (−0.6 MPa) forms and the effect of low positive temperatures on immature plants *S. hybridum* for the first time. The function of the photosynthetic apparatus was studied and a chemical analysis of the level of protective metabolites was conducted. The results of the study demonstrated that, against a background of stressful effects and in the absence of visible morphological changes, there are significant physiological and biochemical changes in plants that are aimed at maintaining the vital activity of plants. The results of assessing the content of the level of secondary metabolites, which are potentially medicinal biologically active substances, can be used in work on the adaptation of *S. hybridum* to adverse natural conditions and for the development of approaches to the targeted synthesis of valuable secondary metabolites as the basis of pharmaceuticals.

## 5. Materials and Methods

### 5.1. Plant Material and Growing Conditions

We studied immature *S. hybridum* plants taken from natural populations in the foothills of the Northern Tien Shan and transferred for the duration of the experiment to growing pots in aquatic culture. The plants had already lost their juvenile characteristics but had not entered the generative period of ontogenesis yet during the immature period of development, when an intensive growth of the shoot was observed. Plants at the time of the experiment were divided into six groups: (1) a control group were grown under 26 ± 3 °C during the day and 20 ± 3 °C at night, with an average air humidity of 37% and optimal irrigation (up to 60% of full moisture capacity); (2) a group subjected to sudden cold treatment at +3 °C in a refrigerator cabinet with lighting (“Polair”, Moscow, Russia) under circadian illumination (using commercial fluorescent white light tubes): 16 h light/8 h darkness regime [200 µmol m^−2^ s^−1^ PAR, light meter LI-205 (Li-Cor, Lincoln, NE, USA)]); (3–4) 2 groups subjected to water deficiency (cultivating in PEG-6000 solution in 200 mM = −0.6 MPa (PEG-1) and 300 mM = −0.9 MPa (PEG-2) concentration) and (5–6) 2 groups subjected to salt stress (cultivating in NaCl solution in 200 mM = −0.6 MPa (NaCl-1) and 300 mM = −0.9 MPa (NaCl-2) concentration). The duration of the stress exposure was 72 h. For the experiment, concentrations of stress agents did not constitute lethal or severe stress, but were regarded as “mild stress” (osmotic potential of the stress agent −0.6 MPa) and “moderate stress” (osmotic potential of the stress agent −0.9 MPa) for this plant species.

The water content (WC) in plant tissues was calculated using the formula:

WC = ((a − b)/a) × 100%,(1)

where a is the initial mass, mg; b is the mass after drying at 105 °C, mg.

### 5.2. Photosynthetic Activity Determination

Photosynthetic activity parameters were estimated by the determination of fluorescence levels. Rapid light curves (RLCs) were recorded using a Junior-PAM (“Heinz WalzGmbH”, Effeltrich, Germany) under actinic illumination of 450 nm. The RLC for each sample was recorded in dark conditions at midnight since *Sedum* is a succulent capable of switching to the CAM-type of photosynthesis under stress conditions [83]. For each measurement, the fluorometer provided eight saturation light pulses of 10,000 µmol/m^2^s every 20 s, while actinic light increased from 0 to 625 µmol m^−2^ s^−1^ gradually [84]. For comparison, the data were obtained from the last pulse of the light curve, when the readings of the light curves of all the parameters under consideration reach a plateau and the difference in indicators is the most objective. The following parameters were calculated using WinControl-3.29 (Walz, Effeltrich, Germany) software: Fv/Fm: maximum quantum yield of PSII photochemistry; Y(II): effective photochemical quantum yield of PSII; Y(NPQ): quantum yield of non-photochemical energy conversion in PSII due to downregulation of the light-harvesting function; Y(NO): quantum yield of non-photochemical energy conversion in PSII that caused by downregulation of the light-harvesting function; PSII relative electron transport (ETR). In the experiment, each time the region of the middle third of the active leaf was selected.

### 5.3. Preparation of Samples for Phytochemical Analysis

10 g of a plant sample of each variant was extracted with 50 mL of 96% ethanol for 30 days. At least three repetitions were prepared for each variant of the experiment.

### 5.4. Qualitative Phytochemical Analysis

Various chemical tests were performed for the presence of biologically active compounds in extracts of *S. hybridum* by using standard methods with minor modifications [85,86,87].

Test for tannins and phenolic compounds: Ferric chloride test: 1–2 drops of a freshly prepared 3% solution of iron (III) chloride were added to 1–2 drops of the extract. A green or blue-green precipitate indicates the presence of phenolic compounds.

Gelatin test: When 1% solution of gelatin was added to the extract, the formation of a white precipitate is observed, indicating the presence of tannins. With an excess of gelatin, the turbidity disappears.

Lead acetate test: 3–5 drops of a 10% solution of basic lead acetate were added to 1 mL of the extract, precipitation from bright-yellow to brown color (hydrolysable tannins (brown precipitate), flavones (brown-yellow precipitate) were observed.

Vanillin–hydrochloric acid test: To 1 mL of the extract, 1–3 drops of a 1% vanillin in concentrated HCl acid were added. The appearance of a range of pink colors indicates resorcinol and phloroglucinol derivatives.

Test for flavonoids: Ammonia test: 1 mL of the extract was mixed with 1 mL of dilute ammonia solution (1%) and then concentrated sulfuric acid was added. A yellow color indicates the presence of flavonoids.

Aluminum chloride test: 1–3 drops of a 1% aluminum chloride solution were added to 1 mL of the extract, an increase in yellow color was observed. The presence of yellow coloration shows the occurrence of flavonoids, all types of polyphenolic compounds with three ordinary OH groups, or OH… S(O)… OH-fragment and flavonol-3-glycosides.

Alkaline reagent test: 2 drops of 5% sodium hydroxide solution were added to 1 mL of extract and a sand-yellow color was observed, it became colorless on the addition of a few drops of diluted acid, which indicates the presence of flavonoids.

Test for quinones: Reaction with an alcohol solution of potassium hydroxide: 3–5 mL of 0.5 N alcohol solution of potassium hydroxide was added to 1 mL of extract. Yellow and green colors were noted. In the air, as it oxidized, the color changed to reddish-brown, indicating different types of connection of dimeric structures of anthraquinones.

Test for alkaloids: Each extract was dissolved in 2 mL dilute HCl in a steam bath and filtered. Two different methods were used. (1) Wagner’s reagent: 1 mL of the above filtrate was treated with a few drops of Wagner’s reagent. A reddish-brown precipitate indicates the presence of alkaloids. (2) Dragendorff’s reagent: a few drops of Dragendoff’s reagent were added to 1 mL of filtrate, the occurrence of orange-red precipitate was taken as positive.

Test for cardiac glycosides: Keller–Killiani test: 1 mL of extract was mixed with 2 mL of glacial acetic acid containing 1–2 drops of ferric chloride solution. The mixture was then poured into another test tube containing 2 mL of concentrated sulfuric acid. A brown ring at the interface indicates the presence of a deoxysugar, characteristic of cardenolides.

Test for terpenoids and/or steroids: Salkowski’s test: 1 mL of extract was mixed in 2 mL of chloroform, and concentrated sulfuric acid was added carefully and shaken gently. A reddish-brown color indicates the presence of a steroidal ring.

Vanillin–sulfuric acid test: To 1 mL of the extract, 1–2 drops of 1% vanillin in concentrated sulfuric acid were added. The appearance of red-violet or purplish-red staining was noted, indicating the presence of terpenes with C3-OH, C3-O-glycosides.

Test for carbohydrates. Molisch’s test: 2 mL of extract was poured into the test tube, 3–4 drops of Molisch’s reagent (α-naphthol in 95% ethanol) were added and shaken, concentrated sulfuric acid was then poured carefully down the side of the test tube. A violet ring at the interphase indicates the presence of carbohydrates.

Test for amino acids. Ninhydrin test: 1 mL of extract and 0.5 mL of 1% ninhydrin solution were poured into the test tube. The contents of the test tube were carefully heated until blue-violet staining appeared. Purple staining of various shades is specific to α amino acids.

### 5.5. Thin Layer Chromatography

Pre-coated silica aluminum sheets (E. Merck, 0.2 mm thick, 20 × 20 cm) were used to perform thin-layer chromatography (TLC) and visualized under UV light of 254 and 365 nm, followed by spraying developers.

The following different solvent systems were used as developing systems: chloroform:methanol:water (4:3:1), n-butanol:acetic acid:water (10:3:7), chloroform: water (1:1), chloroform:methanol:acetone (8:1:1) for flavonoids; n-butanol:acetic acid:water (4:1:1) for terpenoids; hexane:ethyl acetate:methanol:25% ammonia (3:3:1:0.1), hexane:ethyl acetate:methanol:water (6:12:4:1) and ethyl acetate:chloroform:water (5:3:1) for alkaloids [88,89].

The plates were air-dried and visualized by spraying with vanillin-sulfuric acid spray followed by heating for 5 min at 110 °C for detection of steroids and triterpenoids, Dragendorff’s reagent for alkaloids. Flavonoids were detected under ultraviolet rays at 365 nm and aluminum chloride spray and iron (III) chloride spray solution for phenolic compounds.

The R*_f_* value is the identification characteristic in TLC and depends on the used combination of solvents.

### 5.6. Gas Chromatography-Mass Spectrometry Analysis

GC-MS analysis of *S. hybridum extracts* was performed on Agilent 6890N/5973N (Santa Clara, CA, USA). Sample volume 1.0 µL, sample injection temperature 260 °C, without flow division. Separation was carried out using a chromatographic capillary column DB-35MS with a length of 30 m, an inner diameter of 0.25 mm, and a film thickness of 0.25 µm at a constant carrier gas (helium) velocity of 1 mL/min. The chromatographic temperature was programmed from 40 (exposure 0 min) to 150 °C with a heating rate of 10 °C/min (exposure 0 min) and up to 300 °C with a heating rate of 5 °C/min (exposure 10 min). Detection was carried out in the SCANm/z 34–850 mode. Agilent MSDChemStation software (version 1701EA) (Santa Clara, CA, USA) was used to control the gas chromatography system, register and process the obtained results and data. Data processing included determination of retention times, peak areas, as well as processing spectral information obtained using a mass spectrometric detector. The Wiley 7th edition and NIST’02 libraries were used to decode the obtained mass spectra (the total number of spectra in the libraries is more than 550 thousand).

### 5.7. Data Analysis

All experiments were done in three replicates. The processing of data and graphing were performed using Microsoft Excel (Microsoft Corp., Redmond, Washington, DC, USA). Student’s *t*-test was used to analyze the differences between the samples (Statistica 12, StatSoft Inc., Tulsa, OK, USA). Atypical values were excluded from the data based on *t*-tests, the standard error of the average sample was calculated. Differences were considered significant at *p* < 0.05. Data analysis: For quantum yields and RLCs parameters, the analysis of variance (ANOVA) was performed using control data as fixed factors. Plus/minus signs in the tables show the standard error of the mean value. Graphs present mean values with standard error bars. Asterisks * indicate the reliability of results at 0.05 significance level, respectively (unless otherwise is pointed out). Values on diagrams presented are means (±SD). Different letters above the bars represent significant differences at *p* ≤ 0.05, *n* = 3–12 plants in each of 3 replicates for all treatments.

## Figures and Tables

**Table 1:** Plant biomass and water content in plant tissues, biomass accumulation and water content in plant tissues in *S. hybridum* plants under control and stressful conditions. Same letters “^a^” above numerical values represent no significant differences at *p* ≤ 0.05. Values are means ± SE, *n* = 12.

Cultivation Conditions	Average Biomass of 1 Plant, g	Average Water Content in Plant Tissues, %
Control	0.46 ± 0.02 ^a^	99.6
PEG-6000 (1) 200 mmol/L	0.47 ± 0.01 ^a^	99.5
PEG-6000 (2) 300 mmol/L	0.48 ± 0.01 ^a^	99.5
NaCl (1) 200 mmol/L	0.46 ± 0.01 ^a^	99.6
NaCl (2) 300 mmol/L	0.45 ± 0.02 ^a^	99.6
Cold (+3 °C)	0.46 ± 0.02 ^a^	99.6

## Data Availability

Not applicable.

## Supplementary Materials

The following supporting information can be downloaded at: https://www.mdpi.com/article/10.3390/plants11060828/s1, Table S1: Determination of the qualitative composition of the main classes of biologically active compounds; Table S2: Change in content of metabolites in *Sedum hybridum* L. under Mild and Moderate stress conditions.
