# Supplementary material for: The Reactions of Photosynthetic Capacity and Plant Metabolites of Sedum hybridum L. in Response to Mild and Moderate Abiotic Stresses
Source: Plants (Basel). 2022 Mar 21;11(6):828. doi: 10.3390/plants11060828 (PMC8955115; doi:10.3390/plants11060828)
Supplement: Supplementary file 1 [file plants-11-00828-s001.zip › plants-1580443-supplementary.pdf]

Table S1. Determination of the qualitative composition of the main classes of biologically active compounds.

№	Phytochemical test	Samples					
		Control	PEG-6000 (1) 200 mmol /l	PEG-6000 (2) 300 mmol /l	NaCl (1) 200 mmol /l	NaCl (2) 300 mmol /l	Cold (+3 °C)
1	Ferric chloride test (tannins and phenolic compounds)	++	+	+++	+	+++	++
2	Gelatin test (tannins)	++	+	++	+	+	+
3	Lead acetate test (tannins and phenolic compounds)	++	++	++	++	++	++
4	Vanillin–hydrochloric acid test (phenolic compounds)	++	-	+	+	++	+++
5	Ammonia test (flavonoids)	++	+	+	+	++	++
6	Aluminum chloride test (flavonoids)	+++	++	+++	++	+++	++
7	Alkaline reagent test (tannins and flavonoids)	++	++	++	++	++	++
8	0,5 N KOH / alcohol (quinones)	++	+	+++	+	+++	++
9	Wagner's reagent (alkaloids)	-	+++	+++	-	-	-
10	Dragendorff's reagent (alkaloids)	-	+++	+++	-	-	-
11	Keller-Killiani test (cardiac glycosides)	++	++	+++	++	+++	++
12	Salkowski's test (terpenoids and steroids)	+++	+	+	++	+++	++
13	Vanillin–sulfuric acid test (terpenoids and steroids)	+++	+	++	+	++	+++
14	Molisch's test (carbohydrates)	++	++	++	++	++	++
15	Ninhydrin test (amino acids)	+++	++	++	++	+++	+++

The intensity of the analytical effect in the form of color change or precipitation is expressed as follows:

+++ – strong analytical effect;

++ – average analytical effect;

+ – weak analytical effect;

- – no analytical effect

Table S2. Change in content of metabolites in *Sedum hybridum* L. under Mild and Moderate stress conditions.

Retention time, min	Secondary metabolits	Content, %					
		control	PEG-6000 (1)	PEG-6000 (2)	NaCl (1)	NaCl (2)	cold (+3 ◦C)
		fatty acids					
17.55	Erucic acid	0.18	-	-	-	-	-
36.14	Octadecanoic acid	1.96	-	-	-	-	-
		fatty acid esters					
32.50	Hexadecanoic acid, ethyl ester	1.00	1.76	1.98	1.78	0.72	1.10
33.66	Sebacic acid, butyl ethyl ester	-	1.25	0.88	1.03	0.35	0.67
36.19	Octadecanoic acid, ethyl ester	-	2.92	1.30	-	-	1.23
36.19	Linoleic acid ethyl ester	-	-	-	1.70	0.60	-
37.14	Decanedioic acid, dibutyl ester		6.07	5.34	5.41	2.27	5.00
40.26	Ethyl stearate, mono 9-epoxy	0.89	2.14	0.90	2.25	0.45	1.16
		alkane hydrocarbons					
17.38	Tetradecane	0.24	-	-	-	-	-
19.96	Pentadecane	0.33	-	-	-	-	-
22.42	Hexadecane	0.47	-	-	-	-	0.55
24.76	Heptadecane	2.00	1.65	1.05	-	-	-
26.98	Octadecane	-	-	-	-	-	1.11
31.14	Eicosane	0.96	1.62	-	-	-	-
33.08	Heneicosane	2.37	2.83	-	-	-	0.85
34.94	Docosane	2.52	1.95	-	0.99	-	1.62
37.14	Tricosane	4.29	1.96	0.87	1.03	-	2.46
38.43	Tetracosane	3.60	3.84	1.07	-	0.39	2.05
40.07	Pentacosane	-	2.09	-	1.18	-	-
40.08	Hexacosane	3.15	-	-	-	-	2.15
43.20	Heptacosane	2.38	-	0.83	-	-	-
44.67	Tetratetracontane	-	-	-	2.52	0.53	1.35

continuation of Table S2

44.68	Octacosane	2.18	1.60	-	-	-	1.13
46.10	Nonacosane	-	-	1.23	-	-	-
46.11	Hexatriacontane	1.25	-	-	-	-	-
19.34	Cyclohexane, octyl-	0.51	-	-	-	-	-
			terpenes and terpenoids				
18.62	Hexadecane, 2,6,10,14-tetramethyl-	0.59	-	-	-	-	-
20.48	Decahydro-4,4,8,9,10-pentamethylnaphthalene	0.31	-	-	-	-	-
23.20	Pentadecane, 2,6,10,14-tetramethyl-	0.90	-	-	-	-	-
26.75	Hexadecane, 2,6,10,14-tetramethyl-	0.95	-	-	-	-	-
26.99	Octadecane, 3-ethyl-5-(2-ethylbutyl)-	-	0.99	0.48	0.90	0.25	0.22
34.76	Phytol	-	-	1.25	1.44	0.40	1.09
35.11	18-Norabieta-8,11,13-triene	3.39	2.23	-	3.00	0.39	1.60
41.56	Methyl dehydroabietate	-	-	-	-	0.29	1.25
			ubiquinones				
52.01	γ-Tocopherol	-	-	0.65	-	0.46	-
			naphthoquinones				
46.61	2-Anilino-1,4-naphthoquinone	0.95	-	-	-	-	2.34
			amines and their derivatives				
10.11	Aniline	-	-	0.33	0.58	0.16	0.69
28.24	[1,1'-Biphenyl]-2-amine	-	-	1.22	-	-	-
28.27	Diphenylamine	0.78	1.63	-	-	-	-
			aromatic acid				
19.34	5-O-Methyl-d-gluconic acid dimethylamide	-	11.25	-	-	-	-
			organic heterocyclic compounds				
17.90	Benzothiazole	0.63	-	-	-	-	1.30
30.86	2(3H)-Benzothiazolone	-	-	2.05	-	-	-

continuation of Table S2

alcohols and their derivatives							
11.74	Glycerin	-	-	-	-	-	32.00
17.55	1-Hexadecanol, 2-methyl-	-	-	-	-	-	0.21
19.71	S)-(-)-1,2,4-Butanetriol, 2-acetate	-	-	-	30.75	-	-
19.95	1,2,4-Butanetriol	-	-	38.11	1.07	19.18	-
phenolic compounds							
22.27	1,2,3-Benzenetriol	-	-	-	-	2.12	-
25.32	1,4-Benzenediol, 2,5-bis(1,1-dimethylethyl)-	0.90	2.40	1.61	2.71	-	1.03
